# Spatiotemporal variability in a copepod community associated with fluctuations in salinity and trophic state in an artificial brackish reservoir at Saemangeum, South Korea

**DOI:** 10.1371/journal.pone.0209403

**Published:** 2018-12-20

**Authors:** Yusuke Oda, Sho Nakano, Jong-Mo Suh, Hye-Ji Oh, Mei-Yan Jin, Yong-Jae Kim, Masaki Sakamoto, Kwang-Hyeon Chang

**Affiliations:** 1 Department of Applied Environmental Science, Kyung Hee University, Yongin, Gyeonggi-do, Republic of Korea; 2 Department of Life Science, Daejin University, Pocheon, Gyeonggi-do, Republic of Korea; 3 Department of Environmental Engineering, Toyama Prefectural University, Imizu, Toyama, Japan; University of Hyogo, JAPAN

## Abstract

Saemangeum Reservoir in South Korea is an estuarine system enclosed by a dyke construction, where seawater inflow and retained water outflow are managed by the opening/closing of sluice gates installed in the southern part of the dyke. An exchange of the reservoir water can cause spatiotemporal fluctuations in the salinity and trophic state, which are major drivers determining variation in the composition of biological communities in estuarine systems. Here, we investigated the seasonal and spatial variability in the copepod community and environmental conditions (water temperature, salinity, transparency, chlorophyll *a* concentration, total nitrogen, total phosphorus and Carlson’s trophic state index) based on seasonally conducted field monitoring in the Saemangeum Reservoir from July 2013 to January 2018. In addition to the role of temperature, salinity and chlorophyll *a* concentration in structuring the copepod community and diversity, the biological indices of copepods with respect to salinity range and trophic state, were evaluated. The spatiotemporal variability in the salinity and trophic state variables showed contrasting patterns, and chlorophyll *a* concentration was negatively affected by salinity, indicating that the reservoir water was being highly exchanged with opening of the sluice gates. The mean trophic state index values, however, were constant in the eutrophic state (50―70). Dominant copepods were *Acartia* (*A*. *hudsonica*, *A*. *sinjiensis*, *Acartia* spp.) and *Oithona* (*O*. *davisae* and *Oithona* spp.), which are common species in eutrophic neritic water. Variation in the copepod community was mainly associated with the seasonal succession of the dominant species rather than a spatial gradient (from around the estuary to the sluice gates); however, site-specific differences in frequencies of several non-dominant species could be detected around the estuary (*Sinocalanus tenellus*) and the sluice gates (*Centropages* spp., *Tigriopus* spp. and *Labidocera rotunda*). The copepod diversity increased with species-richness from around the estuary to the sluice gates, which could result from variation in the site-specific location of non-dominant species. The frequency of particular species was also able to discriminate in terms of the salinity range (oligohaline: *A*. *pacifica*, *S*. *tenellus* and *A*. *sinjiensis*; mesohaline: *Pseudodiaptomus inopinus*; and polyhaline: *C*. *abdominalis* and *Centropages* spp.) and the trophic state (mesotrophic: *C*. *abdominalis*, *Calanus sinicus* and *Centropages* spp.; and hypereutrophic: *S*. *tenellus*, *P*. *inopinus* and *Sinocalanus* spp.). The findings from this study not only identify the factors determining spatiotemporal variation in the copepod community in the Saemangeum Reservoir, but also expand the applicability of copepods as biological indicators of conditions associated with salinity range and trophic state in other enclosed estuarine systems.

## Introduction

Coastal and estuarine ecosystems provide various ecosystem services of direct benefit to humans (such as fisheries, provision of fish nursery habitats, water filtering and detoxification), so the loss of biodiversity and ecosystem function caused by excessive anthropogenic activities are major concern [1,2]. Given the connection between inland and marine waters, the biological communities in such ecosystems can be dynamically influenced by factors such as eutrophication, chemical pollution and tidal current [3,4]. In order to manage these highly productive ecosystems, an improved understanding of the complex variations in biological communities in response to various stressors has been recognized to be an important task in aquatic ecology.

Planktonic organisms are often used as biological indicators for monitoring the impact of human activities on ecological conditions, because they are particularly sensitive to environmental fluctuations [5]. In coastal and marine environments, copepods are mainly used to predict ecological changes because they appear as the dominant zooplankton group, where they make up over 80–90% of the total zooplankton abundance [6]. In addition, copepods are recognized as a major resource, supporting the abundance of economically important fish in coastal or marine environments due to their central role connecting the energy pathway from primary producers (phytoplankton) to higher trophic levels (fishes) in the food web [7,8,9,10]. The spatiotemporal variations in a copepod community (i.e., species composition and distribution) can reflect the environmental conditions [11,12]. However, in comparison to lotic water systems (e.g., lakes and reservoirs), coastal and marine environments can be dramatically regulated by tidal currents, which make it complicated to detect the factors influencing the composition of copepod communities. The tidal cycle can modify the physicochemical (e.g., water temperature and salinity) and biological conditions (e.g., chlorophyll *a* concentration) in the water; in particular, planktonic organisms are vulnerable to tidal currents [13]. For example, the influence of the Kuroshio current on the seasonal succession, distribution, species composition and diversity of the copepod community has been well studied in Taiwan [1,14,15].

**Fig 1:**
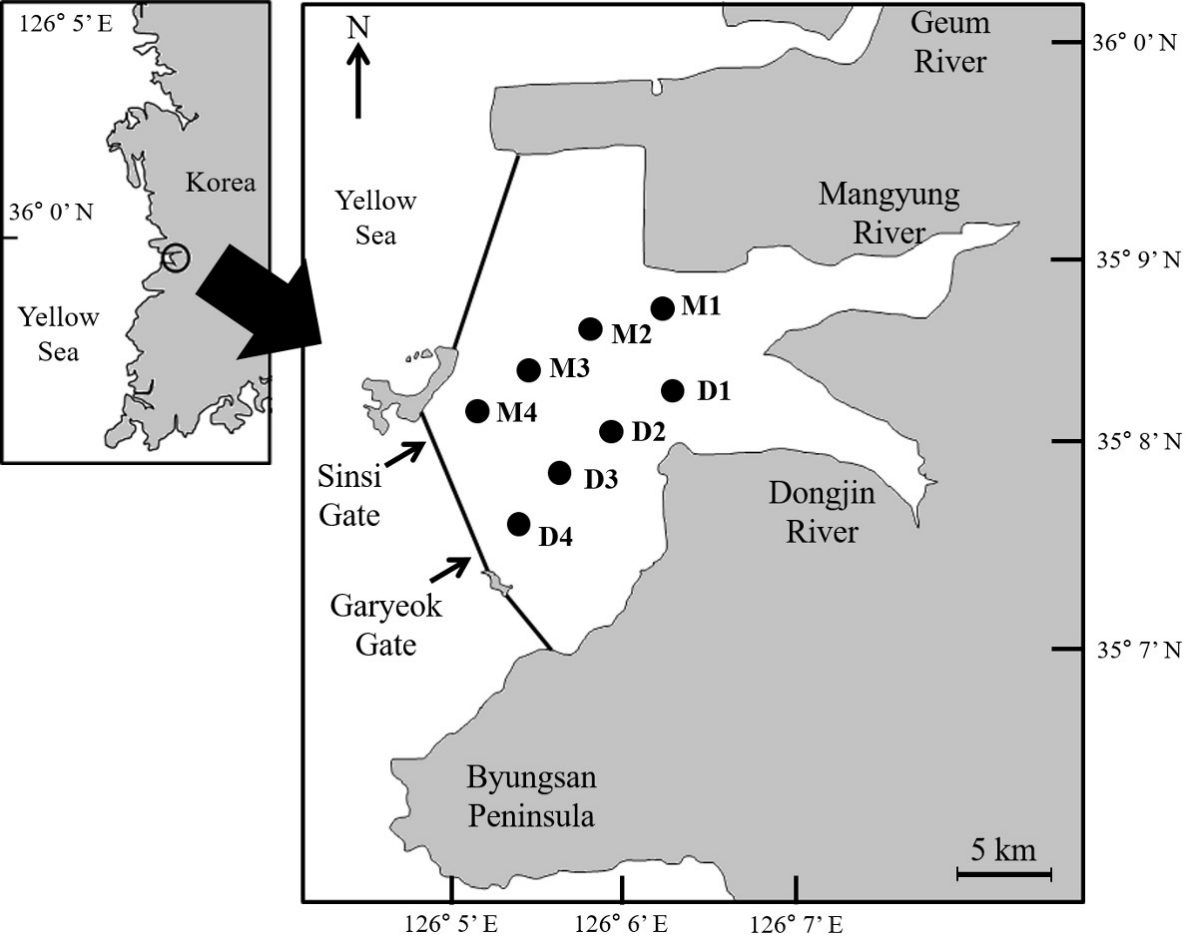
The maps of the Saemangeum Reservoir with eight sampling sites (M1―M4, D1―D4).

The Saemangeum coast is located on the west coast of Korea (Fig 1), where it is connected to the Dongjin and Mangyung Rivers. From 1991 to 2006, a dyke (with a total length of approximately 33 km) was constructed to enclose the inner area of the Saemangeum coast [16], resulting in the creation of a huge brackish reservoir (the Saemangeum Reservoir, with a surface area of 401 km^2^), which is characterized by the mixing of river-discharged freshwater and ocean seawater. The inflow/outflow of seawater and retained water are managed by opening/closing of two sluice gates (the Sinsi- and Garyeok-derived Gates), which were installed in the southern part of dyke [17,18]. Although the dyke was constructed mainly for desalination (improvement of irrigation water supply and quality, and subsequent prevention of salt damage to irrigated land), negative impacts of eutrophication on the aquatic ecosystem, caused by high levels of nutrients entering the reservoir from the rivers, are also becoming apparent (e.g., change in benthic biota [19]). As a provisional countermeasure to eutrophication, the sluice gates are occasionally opened to reduce the deterioration of the water quality. The water replacement can cause dynamic variation in not only the trophic state but also in salinity at both spatial and temporal levels, which could be considerable drivers for change of the plankton community in the Saemangeum Reservoir.

Because tidal currents are largely limited as a result of the dyke [20, 21], changes in the planktonic community in the Saemangeum Reservoir are expected to reflect strongly the environmental conditions. Although the copepod community structure in the Saemangeum Reservoir had been described at both the pre- and post-construction phases of the dyke [22,23], any community variation associated with salinity change and trophic state was not clearly detected. Therefore, the aims of the present study were to evaluate the current environmental conditions of the Saemangeum Reservoir, role of the environmental factors in determining the community structure and diversity of copepods, and the particular species which can be useful as biological indicators of enclosed estuaries associated with particular salinity ranges or trophic states. These were archived by analyzing the spatiotemporal variability of some environmental conditions (water temperature, salinity, transparency, chlorophyll *a* (Chl *a*) concentration, total nitrogen (TN), total phosphorus (TP) and trophic state index (TSI)) and copepod community structure in the Saemangeum Reservoir based on periodic field monitoring data (July 2013―January 2018).

## Materials and methods

### Seasonal field sampling

Field sampling was seasonally (four times a year) conducted at the inner area of the Saemangeum Reservoir (35°49’N, 126°37’E) over 19 months from July 2013 to February 2018 (Fig 1). Eight monitoring sites were selected to cover the reservoir from the area around the estuary (D1 and M1) to the slice gates (D4 and M4); the D and M stand for a line along the Dongjin and Mangyung River, respectively. At each site, zooplankton samples were collected with vertical tows of Kitahara plankton nets (mouth diameter: 30 cm; mesh size: 200 μm) from near the bottom of the reservoir to the surface water. The volume of the filtered water was dependent with the water depth at the sampling site and time. The water depth at each sampling site varied from approximately 2.0 to 35 m. This field study was conducted as a part of a project entitled “Monitoring of Saemangeum Reservoir natural ecosystem,” which was granted permission by Saemangeum Regional Environmental Office, Ministry of Environment.

### Water physico-chemistry analysis

Water transparency was determined using a white Secchi disk (30 cm diameter). Water temperature and salinity were measured from surface water using a multi-parameter water quality monitoring system (Multi-Probe U-20; HORIBA) *in situ*. In addition, surface water was collected to determine TN, TP, and Chl *a* concentration in the laboratory. TN and TP were measured by the ultraviolet spectrophotometer screening method and by the ascorbic acid method, respectively [24]. To measure Chl *a* concentration, the arbitrary volume (200―500 mL) of water samples was filtered with Whatman GF/C filter paper (1.2 μm pore size). After extraction of the pigment by immersing the filter paper in acetone, Chl *a* concentration was calculated from the wavelengths of 630 nm, 663 nm, 645 nm and 750 nm [24]. The trophic state index (TSI) was calculated as the average value of TSI (SD), TSI (TP) and TSI (Chl *a*) according to Carlson’s equations [25] as:

$$\mathrm{TSI}(\mathrm{SD})=10\left(6-\frac{\mathrm{In}(\mathrm{SD})}{\mathrm{In}2}\right)$$

$$\mathrm{TSI}(\mathrm{TP})=10\left(6-\frac{\mathrm{In}\left(\frac{48}{\mathrm{TP}}\right)}{\mathrm{In}2}\right)$$

$$\mathrm{TSI}(\mathrm{Chl}\;a)=10\left(6-\frac{2.04-0.68\mathrm{In}(\mathrm{Chl}\;a)}{\mathrm{In}2}\right)$$

where SD, TP and Chl *a* mean the obtained values of Secchi disk transparency (m), TP (mg m^-3^), and Chl *a* concentration (mg m^-3^), respectively, corresponding to the site and time. The ranges of each TSI were 36.8―77.4 (TSI (SD)), 12.0―95.1 (TSI (TP)), 39.2―83.5 (TSI (Chl *a*)), and 42.5―80.6 (averaged TSI).

### Zooplankton abundance and composition analysis

Zooplankton samples were immediately preserved with seawater containing formalin at a final concentration of 5% (v/v). In the laboratory, adult copepods were identified to genus or species level and counted using a Sedgewick-Rafter Counting chamber under a microscope (× 400 magnification) in 1/5 to 1/10 aliquots of each sample. The species-specific abundance values (Ind m^-3^) were calculated by dividing the individual numbers of each taxon estimated from each sample by the volume of water filtered in the field. Based on the data obtained from the total of 152 (eight sampling sites × 19 sampling dates) samples, mean abundance (*MA*), frequency of occurrence (*FO*) and relative contribution (*RC*) to the total sample by each identified taxon were calculated according to the following equations:

$$MAx=\frac{\sum \left(\mathrm{abundance}\;\mathrm{of}\;x\right)}{152}$$

$$FOx=\frac{\sum \left(\mathrm{occurence}\;\mathrm{of}\;x\right)}{152}$$

$$RCx=\frac{MAx\times FOx}{\sum \left(MAx\times FOx\right)}$$

where *x* denotes the species. The dominant rank in the copepod species present was determined based on the relative contributions of the various species. Total abundance, number of species, Margalef’s species richness (*D´*), Pielou’s evenness (*J´*) and Shannon-Wiener diversity (*H’*) indices were also calculated for each sample using Primer 6 software (ver. 6. 1. 16).

### Data analysis

The seasonal difference and spatial gradient (D1―M1, D2―M2, D3―M3 and D4―M4) in the environmental parameters (water temperature, transparency, salinity, Chl *a* concentration, TN, TP and TSI) and the community descriptors (total abundance, number of species, richness, evenness and diversity indices) were determined with a linear mixed-effect model [26] as generated variance in “season against spatial gradient” or “spatial gradient against season,” to remove the random effect. After applying the one-way analysis of variance (ANOVA) to the models to which each factor was fitted, multiple comparisons in the form of the post-hoc Tukey–Kramer test were conducted for the groups summarized as “season” or “spatial gradient.” The *P*-values were adjusted with Holm’s method, to control the familywise error rate associated with multiple comparisons. The linear mixed-effects model and the Tukey–Kramer test were performed using “lme4” and “multcomp” packages, respectively, in R program version 3. 3. 1 (R Development Core Team, Vienna, Austria (http://www.R-project.org/)).

The inter-variations in the copepod community and their association with environmental factors (water temperature, salinity and Chl *a* concentration) were described with a non-metric multidimensional scaling (NMDS) ordination to the community metrics converted to Bray-Curtis dissimilarity using the vegan 2.5–2 package in R. The environmental factors were fitted onto the ordination plots of the copepod community if the probability values were less than 0.05, in which the length and vectors of environmental factors were resulted from correlation analysis between the community- and environment metrics, using the “envfit” function. We also conducted permutation (1000 times) multivariate analysis of variance (PERMANOVA, [27]) on the dissimilarities with “adonis” function to test the differences in copepod community in terms of groups season (spring, summer, fall and winter), site (the spatial gradient), salinity range (oligohaline: 0.5―5.0 practical salinity unit (PSU); mesohaline: 5.0―18.0 PSU; polyhaline: 18.0―30.0 PSU) or trophic state (mesotrophic: 40 ≤ TSI < 50; eutrophic: 50 ≤ TSI < 70; hypereutrophic: 70 ≤ TSI). Pairwise comparisons among the group levels were performed with *P*-value corrections of Holm’s method using the “pairwise.perm.anova” function in “RVAideMemoire” package. In addition, species characteristic of the individual community groups, as represented by a product of the “specificity” (how prominent is the mean abundance of the species compared to other groups) and “fidelity” (how prominent the species appears to be in the group), were determined using an indicator value method [28], using the “indval” function in the “labdsv” package.

We applied a generalized linear model (GLM) to evaluate the effects of environmental factors (water temperature, salinity, TN and TP) on Chl *a* concentration, as well as the influence of the environmental factors with Chl a on the copepod abundance of the dominant species (following the species rank), the characteristic species, and the community descriptors. TN and TP were only linked to the model on Chl *a* concentration as an influential factor. The log-link and Gaussian distribution functions were used on all the models. The explanatory variables incorporated into the model were selected using the backward elimination method based on Akaike’s Information Criterion (AIC [29]) to improve the accuracy of GLM analysis. We performed the GLM analysis and the explanatory variable selection with “glm” and “step” functions, respectively.

## Results

**Fig 2:**
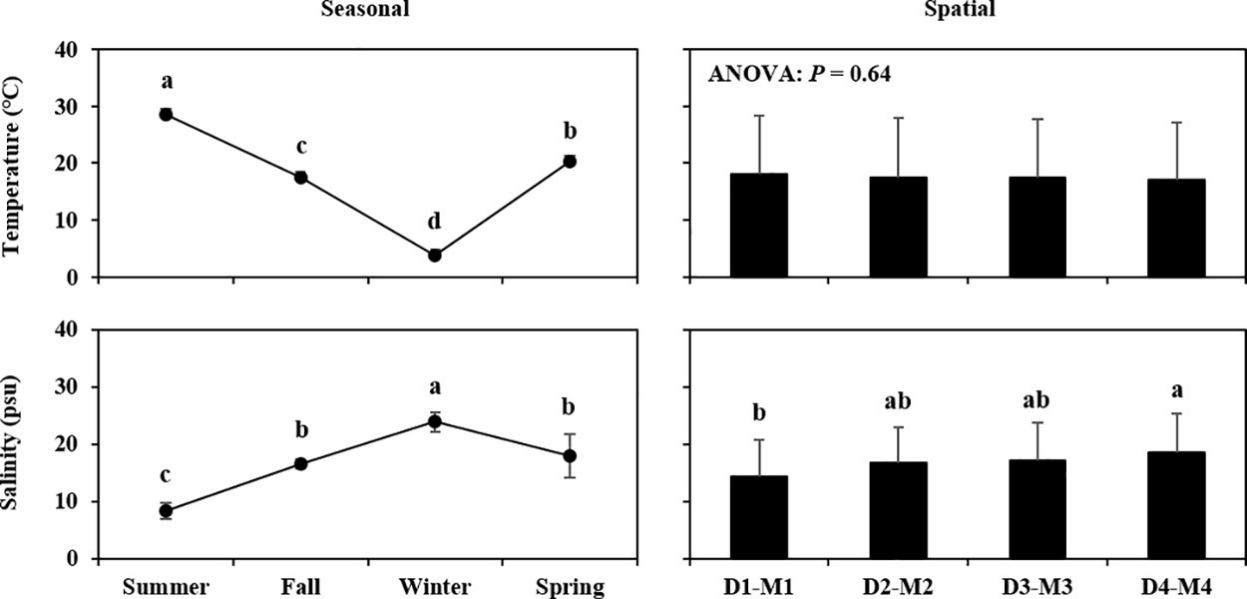
Seasonal and spatial variations in water temperature and salinity. Values are presented as mean ± SD, with the *P*-value indicating the result of one-way ANOVA on the linear mixed-effects model. Samples with a common letter in the figure are not significantly different from each other (*P* > 0.05), following multiple comparison by the Tukey–Kramer test.

**Fig 3:**
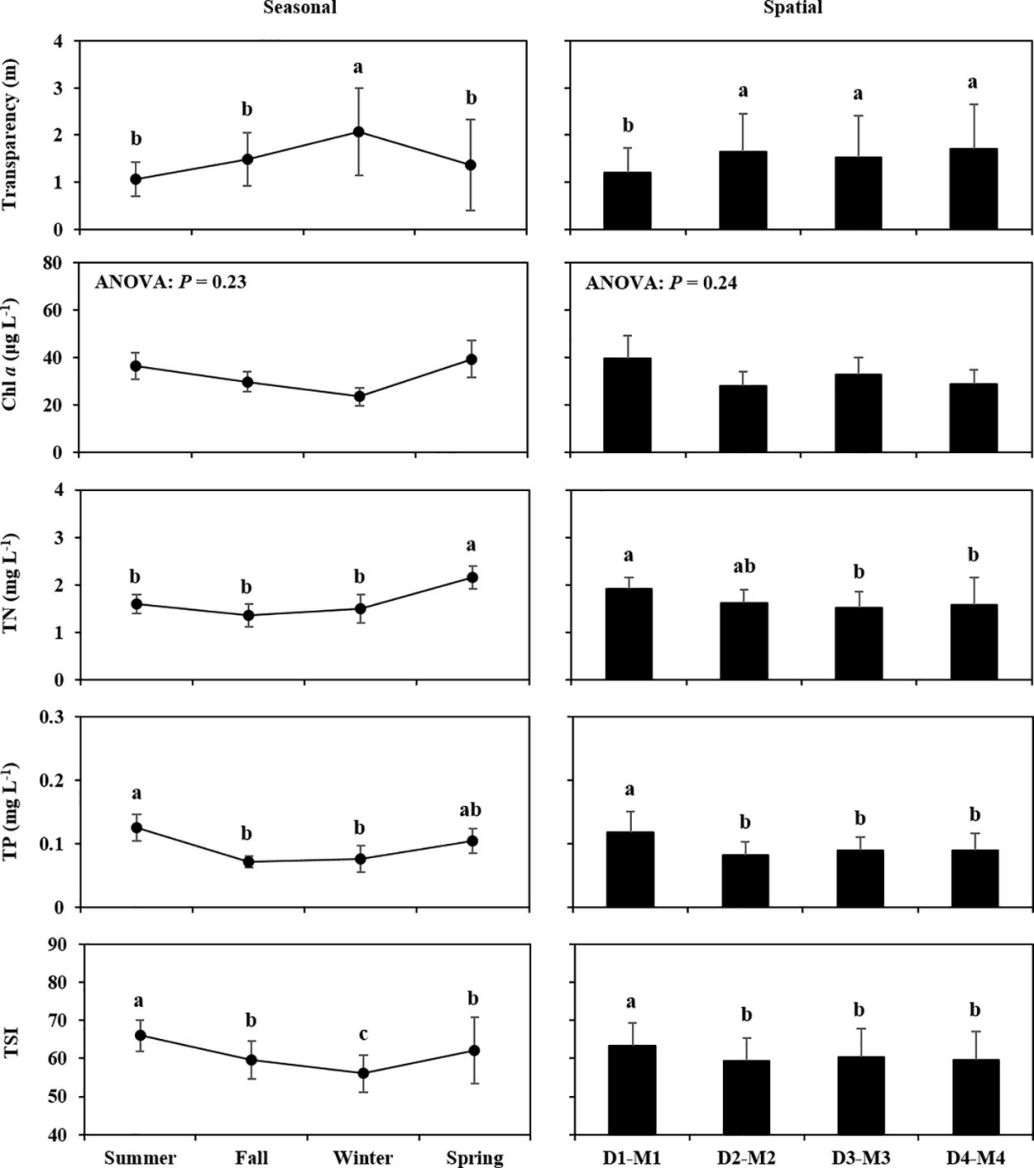
Seasonal and spatial variations in variables related with trophic state (transparency, Chl *a* concentration, TN, TP and the TSI). Values are presented as mean ± SD, with the *P*-value indicating the result of one-way ANOVA on the linear mixed-effects model. Samples with a common letter in the figure are not significantly different from each other (*P* > 0.05), following multiple comparison by the Tukey–Kramer test.

The seasonal variations and spatial gradients in water temperature, salinity, and the other parameters related to trophic state (transparency, Chl *a* concentration, TN, TP and TSI) are shown in Fig 2 and Fig 3, as the mean values with standard deviation (SD) (mean ± SD). Where the results of the one-way ANOVA on the linear mixed-effect model fitted to the environmental parameters were significant at the *P* < 0.05 probability level, the results of the Tukey–Kramer test to each group (“Season” or “Spatial”) are shown on the figures.

Water temperature strongly reflected seasonal patterns, with maximum mean ± SD values in summer (28.63 ± 0.56°C) and minimum values in winter (3.84 ± 0.38°C). Salinity significantly varied at both the temporal scale (summer: 8.37 ± 1.42 PSU; winter: 23.82 ± 1.66 PSU) and the spatial scale (D1―M1: 14.33 ± 6.35 PSU; D4―M4: 18.54 ± 6.70 PSU). The seasonal fluctuation in salinity contrasted with that of water temperature. Transparency was significantly higher in winter (2.06 ± 0.93 m) and lower in D1―M1 (1.20 ± 0.52 m) than in summer and D4—M4, respectively. On the other hand, although Chl *a* concentration showed similar variation with that of transparency, we did not detect any significant differences in Chl *a* for either the seasonal or the spatial gradients. Nutrient concentrations were highest in different seasons, such as TN (spring: 2.16 ± 0.23 mg L^-1^) and TP (summer: 0.12 ± 0.02 mg L^-1^), while the spatial patterns of nutrients showed highest concentrations on the estuarine side (TN: 1.91 ± 0.25 mg L^-1^: TP: 0.12 ± 0.03 mg L^-1^). The TSI showed significantly higher values in summer (65.95 ± 4.09) and in D1―M1 (63.34 ± 6.09). On the other hand, the range of all the averaged TSI values could be classified as “eutrophic” (TSI value: 50–70) according to Carlson and Simpson [30]. Overall, the environmental parameters showed greater changes in the temporal scale than in the spatial gradient.

**Fig 4:**
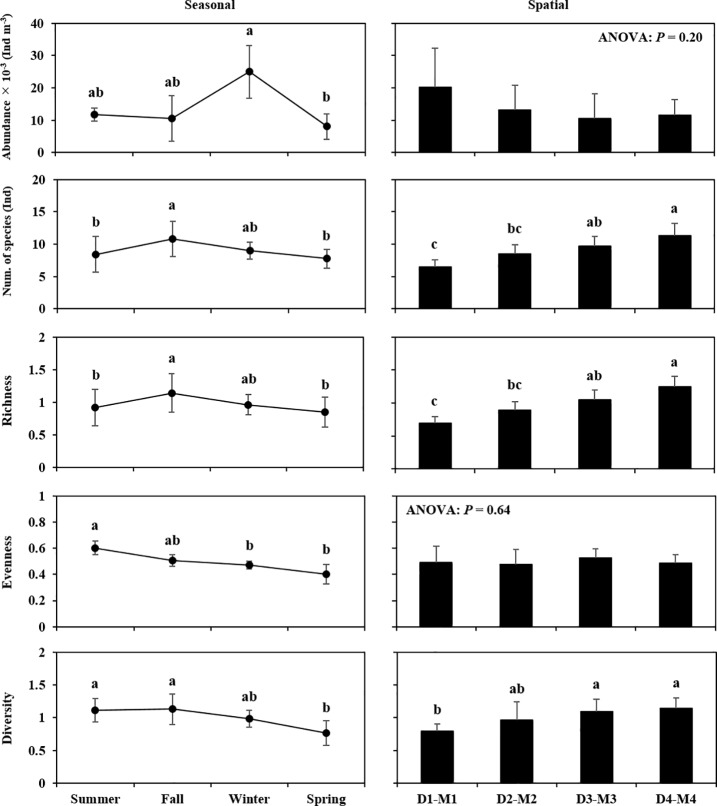
Seasonal and spatial variation in the community descriptors of the copepod community (total abundance, number of species, richness, evenness and diversity index) in the Saemangeum Reservoir. Values are presented as mean ± SD, with the *P*-value indicating the result of one-way ANOVA on the linear mixed-effects model. Samples with a common letter in the figure are not significantly different from each other (*P* > 0.05), following multiple comparison by the Tukey–Kramer test.

The spatiotemporal differences in the community descriptors, namely total abundance, number of species, and in the richness, evenness, and diversity indices, of the copepod community are presented in Fig 4. Total abundance did not show a significant difference over the spatial gradient, but it was much higher in winter ((24.94 ± 8.25) × 10^−3^ Ind m^-3^) than in the summer. The number of species seasonally changed from approximately seven (in spring) to 11 species (in fall), and was likely to be higher around sluice gates (9―14 species) than around estuarine sides (four to eight species). The variations in the species richness index were highly consistent with the number of species in both the seasonal and spatial gradients. The evenness index showed no difference with respect to the spatial gradient, but it showed temporal variations, its value being highest in summer (0.60 ± 0.05) and lowest in spring (0.40 ± 0.08). The diversity index showed the lowest values in spring (0.77 ± 0.19) and in D1―M1 (0.79 ± 0.11).

**Fig 5:**
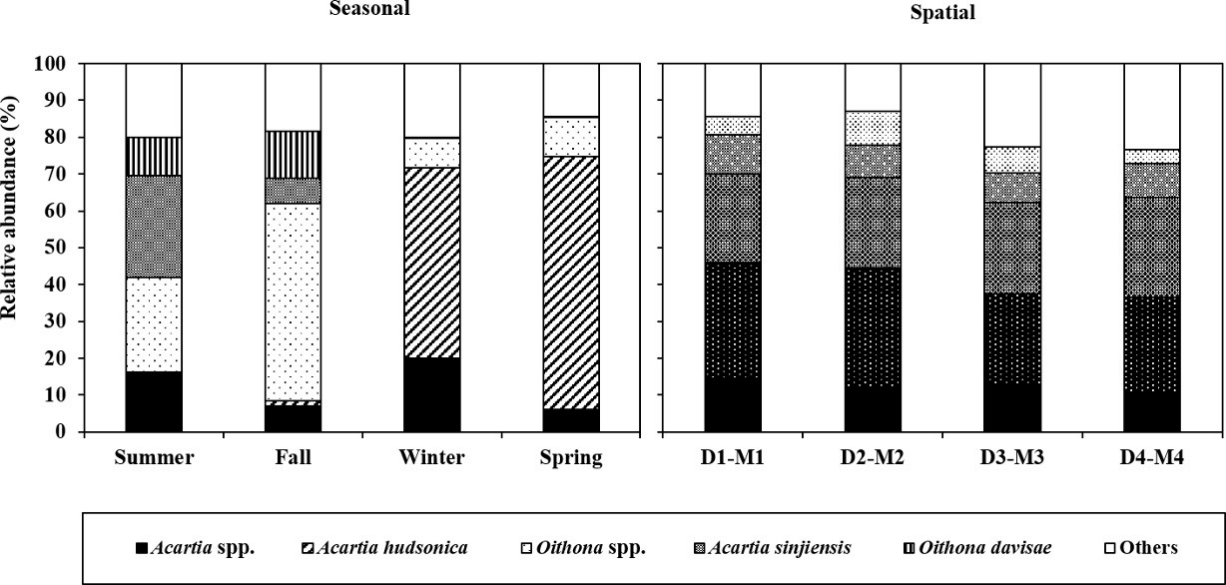
Seasonal and spatial differences in the relative abundance (%) for five dominant species (*Acartia* spp., *A*. *hudsonica*, *Oithona* spp., *A*. *sinjiensis* and *O*. *davisae*) with the sum of abundances of the other species.

The copepod taxa identified in the Saemangeum Reservoir were summarized and characterized according to their dominant rank, mean abundance (*MA*), frequency of occurrence (*FO*) and relative contribution (*RC*) with respect to the community throughout the investigated periods (Table 1). Out of 36 taxa, the five most dominant species (*Acartia* spp., *A*. *hudsonica*, *Oithona* spp., *A*. *sinjiensis* and *O*. *davisae*) represented > 96% of the copepod community. The dominance of these species is likely due to their high mean abundance (approximately > 1,000 Ind m^-3^). In addition to the highly dominant species, *Paracalanus purvus s*. *l*., *Paracalanus* spp., *Sinicalanus tenellus* and *Calanus sinicus* showed relatively high frequencies of occurrence (> 40%). The changes in relative species abundance for the five dominant species and the sum of the others for each season and across the spatial gradient are shown in Fig 5. While the relative abundance exhibited no large difference across the spatial gradient, several changes in the species composition were clearly seen across the seasonal pattern; 1) *A*. *hudsonica* appeared with high dominance from winter to spring, 2) *A*. *sinjiensis* frequently dominated in summer, and 3) *Oithona* spp. and *O*. *davisae* occurred at high frequencies only from summer to fall.

**Table 1:** The dominance rank, mean abundance (*MA*, × 10^−3^ Ind m^-3^), frequency of occurrence (*FO*, %) and relative contributions (*RC*, %) to the total abundance obtained for the 36 copepod taxa identified at eight sampling sites in the Saemangeum Reservoir over 19 months.

Rank	Taxa	*MA* (× 10^3^ Ind m^-3^)	*FO* (%)	*RC* (%)
1	*Acartia* spp.	4.05	84.21	37.52
2	*Acartia hudsonica*	4.47	63.82	31.34
3	*Oithona* spp.	2.07	75.66	17.20
4	*Acartia sinjiensis*	1.44	42.11	6.69
5	*Oithona davisae*	0.95	32.24	3.37
6	*Paracalanus parvus s*.*l*.	0.21	46.71	1.08
7	*Paracalanus* spp.	0.20	42.11	0.91
8	*Oithona similis*	0.11	24.34	0.29
9	*Sinocalanus tenellus*	0.05	46.71	0.28
10	*Corycaeus* spp.	0.05	30.92	0.17
11	*Pseudodiaptomus marinus*	0.07	21.05	0.16
12	*Paracalanus elegans*	0.14	10.53	0.16
13	*Centropages abdominalis*	0.04	25.66	0.13
14	*Centropages* spp.	0.04	28.29	0.12
15	*Eurytemora pacifica*	0.04	26.97	0.12
16	*Pseudodiaptomus inopinus*	0.02	28.95	< 0.01
17	*Calanus sinicus*	0.01	47.37	< 0.01
18	*Calanus* spp.	0.02	35.53	< 0.01
19	*Centropages tenuiremis*	0.02	29.61	< 0.01
20	*Pseudodiaptomus* spp.	0.02	25.66	< 0.01
21	*Eurytemora* spp.	0.02	15.79	< 0.01
22	*Acartia pacifica*	0.01	18.42	< 0.01
23	*Sinocalanus* spp.	0.01	18.42	< 0.01
24	*Labidocera euchaeta*	0.01	13.16	< 0.01
25	*Hemicyclops* spp.	0.01	14.47	< 0.01
26	*Amphiascopsis cinctus*	0.03	5.26	< 0.01
27	*Tigriopus* spp.	< 0.01	4.61	< 0.01
28	*Tortanaus forcipatus*	< 0.01	17.11	< 0.01
29	*Oncaea venusta*	< 0.01	3.29	< 0.01
30	*Labidocera* spp.	< 0.01	9.21	< 0.01
31	*Labidocera rotunda*	< 0.01	7.24	< 0.01
32	*Paracalanus aculeatus*	< 0.01	3.29	< 0.01
33	*Tortanaus* spp.	< 0.01	3.29	< 0.01
34	*Calanopia thompsoni*	< 0.01	2.63	< 0.01
35	*Acartia hongi*	< 0.01	1.32	< 0.01
36	*Temora turbinata*	< 0.01	0.66	< 0.01

**Fig 6:**
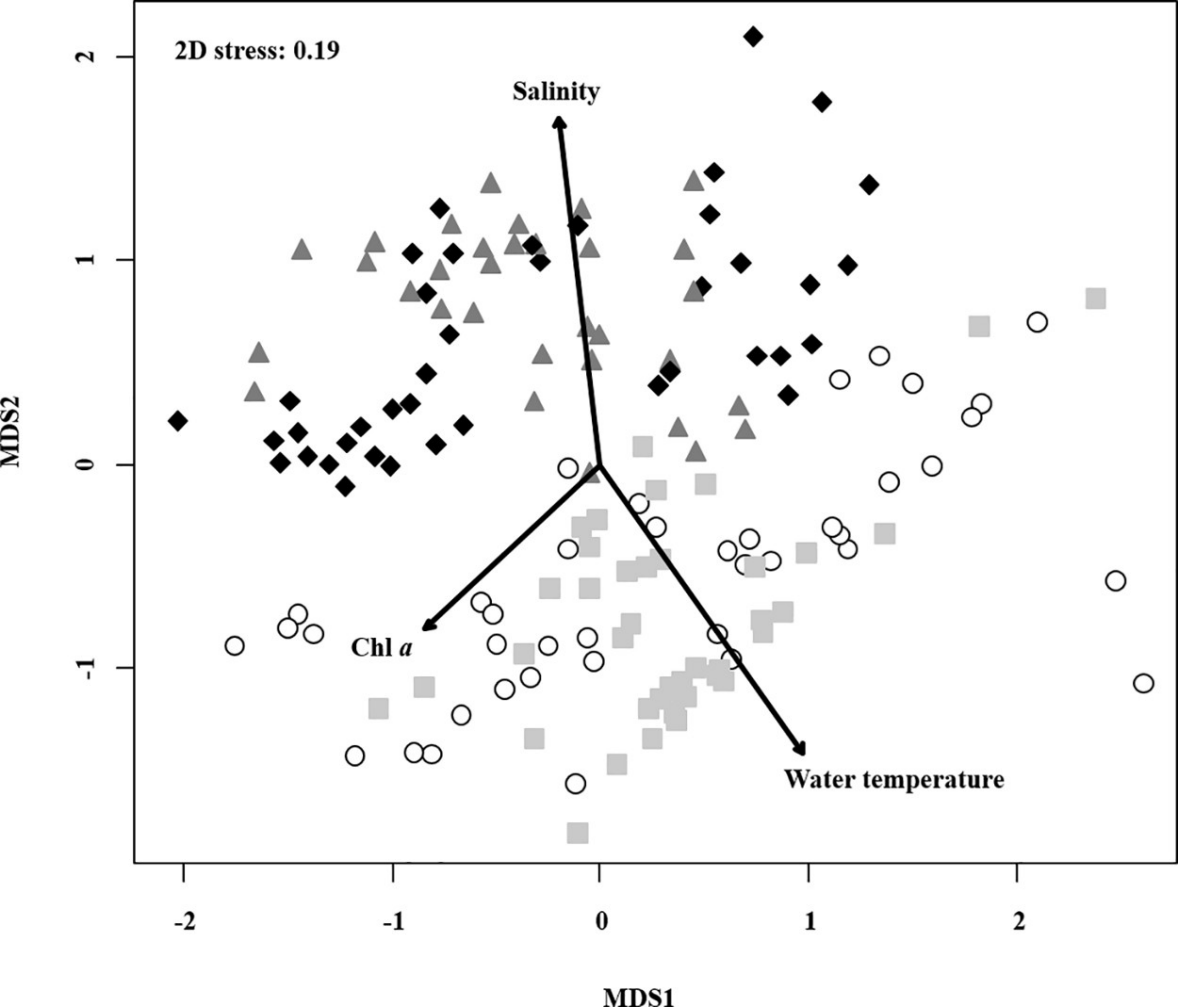
Non-metric multidimensional scaling (NMDS) ordination plot based on the Bray-Curtis dissimilarity on the copepod community. The shape and color of the plotted points divide the community into season; white circle: summer, light-grey square: fall, deep-grey triangle: spring, and black diamond: winter. The arrows indicate the environmental variables (water temperature, salinity, and Chl *a*) which were identified as a significant factor fitting the NMDS ordination (*P* < 0.05). The length and direction of the arrows represent the effect level and the line determining the characteristics of the plots assembly.

The NMDS ordination showed the difference in composition of the copepod community corresponding to a seasonal pattern (Fig 6). In particular, the plots aggregated as “summer and fall” or “winter and spring” could be clearly separated. The environmental parameters (water temperature, salinity, and Chl *a*) fitted onto the NMDS ordination plots were all selected as influential factors (*P*-value < 0.05) on the copepod community. Water temperature and salinity showed longer arrows than Chl *a*, meaning that those factors could have relatively stronger effects in determining the copepod community. In addition, while the directions of all the arrows were largely different, those associated with water temperature and Chl *a* were in directions totally opposite to that of salinity. The results of PERMANOVA on the copepod community showed the probability level to be less than 0.05 except for “trophic state” (Table 2). However, in the pairwise comparison on PERMANOVA, we detected significant differences only among the separation of “season” or “salinity range”. The results indicate that the composition of the copepod community was largely different for each season but could also be changed with respect to the salinity range.

**Table 2:** Pseudo *F*-statistics with the *P*-value generated by PERMANOVA on the copepod community of Bray-Curtis dissimilarity and its pairwise comparison based on Holm's method.

Factors	Pseudo-*F* value	*P*-value	Separation
Season	21.9	< 0.01	Summer―Spring**
			Summer―Fall**
			Summer―Winter**
			Spring―Fall**
			Spring―Winter**
			Fall―Winter**
Site	2.05	< 0.05	DM1―DM2
			DM1―DM3
			DM1―DM4
			DM2―DM3
			DM2―DM4
			DM3―DM4
Salinity range	3.77	< 0.01	Oligoharine―Mesoharine
			Oligoharine―Polyharine*
			Mesoharine―Polyharine**
Trophic state	1.72	0.06	Mesotrophic―Eutrophic
			Mesotrophic―Hypereutrophic
			Eutrophic―Hypereutrophic

The copepod community is separated as four groups: "Season (summer, fall, winter and spring)", "Site (DM1, DM2, DM3 and DM4)", "Salinity range (oligohaline, mesohaline and polyhaline)" or "Trophic state (mesotrophic, eutrophic and hypereutrophic)".

DMx means the combined sampling site Dx and Mx.

Significant differences among the separation are shown in bold.

* *P* < 0.05.

** *P* < 0.01.

Table 3 shows the result of the indicator species analysis (indicator value method) for only three species which were selected to be the most representative species for each group by having higher indicator values. For the groups “season”, “site” and “trophic state”, the characteristic species were not selected at “spring”, “D2―M2”, “D3―M3”, or “eutrophic”. The selected species with Indval values exceeding 50% included *A*. *sinjiensis* (summer; 61.8%), *Oithona* spp. (fall; 66.4%), *Acartia* spp. (winter; 65.6%), *E*. *pacifica* (winter; 63.6%), *A*. *hudsonica* (winter; 63.1%), *A*. *pacifica* (oligohaline; 62.3%), *S*. *tenellus* (oligohaline; 58.3%, hypereutrophic; 78.7%), *C*. *abdominalis* (mesotrophic; 81.6%), *C*. *sinicus* (mesotrophic; 73.1%), and *Centropages* spp. (mesotrophic; 62.2%).

**Table 3:** The groups separated by “Season”, “Site”, “Salinity range” and “Trophic state”, and the three most characteristic species in the selected species identified by the IndVal method with Indval value (%).

Factor	Group	Taxa	Indval value (%)
Season	Summer	*A*. *sinjiensis*	61.8**
		*A*. *pacifica*	49.4**
		*P*. *marinus*	33.6**
	Fall	*Oithona* spp.	66.4**
		*P*. *parvus*. *s*. *l*.	44.2**
		*Paracalanus* spp.	36.2**
	Winter	*Acartia* spp.	65.6**
		*E*. *pacifica*	63.6**
		*A*. *hudsonica*	63.1**
Site	D1―M1	*S*. *tenellus*	30.9*
	D4―M4	*Centropages* spp.	40.5**
		*Tigriopus* spp.	11.9*
		*L*. *rotunda*	11.0*
Salinity range	Oligohaline	*A*. *pacifica*	62.3**
	(0.5―5.0 psu)	*S*. *tenellus*	58.3**
		*A*. *sinjiensis*	41.0*
	Mesohaline	*P*. *inopinus*	37.4*
	(5.0―18.0 psu)		
	Polyhaline	*C*. *abdominalis*	40.9**
	(18.0―30.0 psu)	*Centropages* spp.	38.6*
Trophic state	Mesotrophic	*C*. *abdominalis*	81.6**
	(TSI: 40―50)	*C*. *sinicus*	73.1**
		*Centropages* spp.	62.2**
	Hypereutrophic	*S*. *tenellus*	78.7**
	(TSI: 70―100)	*P*. *inopinus*	46.1**
		*Sinocalanus* spp.	38.0*

* *P* < 0.05.

** *P* < 0.01.

The results of GLM analysis (best model selected by the backward-elimination method) are summarized in Table 4. Water temperature and salinity were negative factors with respect to Chl *a*, but TN had a positive effect. Water temperature was negatively associated with most of the species, including the dominant (except for *A*. *sinjiensis*) and characteristic species (except for *L*. *rotunda*). Total abundance and numbers of species were also negatively affected by water temperature. Salinity showed a positive relationship with only *C*. *abdominalis*, while 11 species were negatively associated with salinity. Total abundance and the evenness index were negatively affected by salinity. Chl *a* had a positive influence on three dominant species (*A*. *sinjiensis*, *O*. *davisae* and *Oithona* spp.) and on the characteristic species *L*. *rotunda*. Although Chl *a* showed a positive relationship with the number of species and with richness index, it had a negative relationship with the evenness index.

**Table 4:** Parameter estimates (±SE) of the generalized linear model (best model).

		Explanatory variables				
Response variables		Water temperature	Salinity	Chl. *a*	TN	TP
Chl. *a*		-1.724 (0.419)***	-3.488 (0.530)***	―	6.790 (3.709)	EX
Dominant species						
	*A*. *hudsonica*	-455.250 (87.990)***	EX	EX	―	―
	*A*. *sinjiensis*	104.372 (43.201)*	EX	28.274 (9.577)**	―	―
	*Acartia* spp.	-766.600 (204.500)***	-665.900 (254.500)**	EX	―	―
	*O*. *davisae*	EX	-92.099 (48.418)	19.149 (8.626)*	―	―
	*Oithona* spp.	EX	EX	20.299 (9.837)*	―	―
Characteristic species						
	*A*. *pacifica*	EX	-2.734 (1.050)*	EX	―	―
	*C*. *sinicus*	-2.004 (0.469)***	-0.991 (0.584)	EX	―	―
	*C*. *abdominalis*	-4.975 (2.700)	5.609 (3.360)	EX	―	―
	*Centropages* spp.	-6.831 (2.040)**	EX	EX	―	―
	*E*. *pacifica*	-9.173 (1.386)***	-5.736 (1.724)**	EX	―	―
	*L*. *rotunda*	0.032 (0.014)*	EX	0.007 (0.003)*	―	―
	*P*. *parvus s*. *l*.	-8.977 (5.585)	-14.196 (6.949)*	EX	―	―
	*Paracalanus* spp.	EX	-14.155 (8.846)	EX	―	―
	*P*. *inopinus*	-3.731 (1.265)**	-5.215 (1.574)**	EX	―	―
	*P*. *marinus*	EX	-13.647 (5.381)*	EX	―	―
	*S*. *tenellus*	EX	-5.872 (1.744)***	EX	―	―
	*Sinocalanus* spp.	EX	-1.661 (0.870)	EX	―	―
	*Tigriopus* spp.	-1.005 (0.665)	EX	EX	―	―
Community discriptor						
	Total abundance	-1364.800 (295.900)***	-1328.700 (368.200)***	EX	―	―
	Number of species	-0.066 (0.033)*	EX	0.029 (0.007)***	―	―
	Richness index	EX	EX	0.002 (0.001)*	―	―
	Evenness index	EX	-0.007 (0.002)**	-0.001 (0.0004)**	―	―
	Diversity index	EX	EX	EX	―	―

EX: excluded parameter by the model selection based on the Akaike Information Criterion (AIC) value.

* *P* < 0.05.

** *P* < 0.01.

*** *P* < 0.001.

## Discussion

In comparison to general coastal environments, the Saemangeum Reservoir has specific conditions characteristic of longer residence of river-discharged freshwater and an occasional water exchange with seawater by the sluice gate operation [18]. The exchange of residence water can prevent the development of a eutrophication process in the Saemangeum Reservoir, as had already been suggested in similar systems, such as Lake Grevelingen in the Netherlands [31], the Shihwa Lake in Korea [32] and Isahaya Bay in Japan [33]. In the present study, we observed contrasting pattern between salinity and Chl *a* concentration (Fig 2, Fig 3), as well as the negative effect of salinity on Chl *a* concentration (Table 4), implying that the residence water could be regularly diluted with the gate opening. In contrast, the increase of the trophic state related parameters during spring to summer (Fig 3) can reflect on the seasonal precipitation and watershed runoff [21]. On the other hand, the the averaged TSI values indicated that the Saemangeum Reservoir is almost eutrophicated at both temporal and spatial scale (Fig 3; [30]), implying that the gate operation might have minor role in regulating the trophic condition. Following the completion of Saemangeum dyke construction, the tidal regime (range and currents) at the Saemangeum Reservoir were markedly limited (more than 80%) even though the sluice gates were fully opened [20]. In addition, the seawater exchange ratio (flushing rate, %) within the impoundment after completion of the dyke was decreased to half of the dyke construction stage at 4.5 km opening [21]. Although we have no available information supporting the level of water exchange at the Saemangeum Reservoir for our monitoring period, the present results and these previous works suggest that the current trophic conditions at the Saemangeum Reservoir are maintained as being eutrophicated despite of the occasional gate operation.

In 2004―2005 and 2007―2008, the periods pre- and post-construction of the Seamangeum dykes, respectively, several copepod species had been reported to dominate zooplankton communities in the Saemangeum Reservoir [22,23]. *A*. *hongi*, *P*. *indicus* and *P*. *parvus s*. *l*. were identified as the dominant species in both reports, though these species were not particularly abundant in our investigations (Table 1). Instead of that, we confirmed some *Acartia* (*A*. *hudsonica*, *A*. *sinjiensis* and *Acartia* spp.) and *Oithona* (*O*. *davisae* and *Oithona* spp.) species to be highly dominant, which are well known to occur abundantly in eutrophicated neritic water of coastal system [34,35]. Therefore, the replacement of the dominant copepod species in the Saemangeum Reservoir could be strongly associated with eutrophication. The occurrence of *Oithona* species may especially represent the consequence of eutrophication: the egg-sac carrying cyclopoid species can maintain its population growth regardless of hypoxia-induced mortality [36]. Another reason for the change in dominant species could be a bottom-up effect in which resource availability for *Acartia* and *Oithona* may not simply be limited by phytoplankton biomass. Several studies have suggested that *Acartia* and *Oithona* can utilize not only phytoplankton but also microzooplankton (e.g., protozoans and ciliates) as a significant source of nutrition [37]. Thus, the present environmental conditions in the Saemangeum Reservoir may provide a habitat favorable to *Acartia* and *Oithona*.

The occurrence of dominant copepod species showed a species-specific seasonal change (Fig 5), with *A*. *hudsonica* being particularly abundant in winter-spring, *A*. *sinjiensis* in summer, and *O*. *davisae* and *Oithona* spp. in summer-fall. The seasonal pattern observed in the current study in *A*. *hudsonica* is highly consistent with findings from many previous studies, which had been explained by the observation that resting periods of *A*. *hudsonica* start in response to temperatures above 15°C [38]. Milione and Zeng [39] experimentally investigated the effects of temperature and salinity on population growth and egg hatching success of *A*. *sinjiensis*, and concluded that the most favorable conditions for *A*. *sinjiensis* are 30°C and 30 PSU salinity. In addition, the authors also found that the hatching rate of resting eggs of *A*. *sinjiensis* did not differ along the gradient 10―50 PSU salinity. Because we observed high densities of *A*. *sinjiensis* in summer regardless of the low prevailing salinity (8.37 ± 1.42 PSU), the appearance of *A*. *sinjiensis* might be triggered by high temperatures rather than high salinity. For *Oithona*, the egg production rate has been known to be extremely low from winter to spring, but to increase greatly in May to June [34]. In addition, given that the potential growth rates of the nauplii and copepodites were highest in the summer [40], the seasonal pattern observed in *Oithona* is not surprising. The result of NMDS ordination indicated that the composition of the copepod community could be very different if the plot assembly was identified as “summer and fall” and “winter and spring” (Fig 6). This phenomenon could be strongly related to the seasonal change in dominant species (winter and spring: *A*. *hudsonica*; summer and fall: *A*. *sinjiensis*, *Oithona* spp. and *O*. *davisae*; Fig 5). On the other hand, the PERMANOVA on the Bray-Curtis dissimilarity of the copepod community detected significant differences among all seasons (Table 2). Except for spring, high-frequency species for each season could be characterized with not only dominant species but also non-dominant species (Table 3): summer (*A*. *sinjiensis*, *A*. *pacifica* and *P*. *marinus*), fall (*Oithona* spp., *P*. *parvus s*. *l*. and *Paracalanus* spp.), winter (*Acartia* spp., *E*. *pacifica* and *A*. *hudsonica*). The seasonally different occurrences of the non-dominant species agreed closely with previous literature [7,41]. Thus, the composition of the copepod community can be variable, with changes associated with seasonality, but the variation would clearly appear to lie between “summer and fall” and “winter and spring” due to the replacement of the dominant species.

Although the spatial difference in composition of the copepod community was not as clear as the seasonal difference, we could identify characteristic species around the estuary sides (*S*. *tenellus*) and around the sluice gates (*Centropages* spp., *Tigriopus* spp. and *L*. *rotunda*) (Table 3). The differences in distribution among these species may be related to the spatial gradient of salinity level and/or trophic status (Fig 2, Fig 3, Table 3). Uye et al. [7] reported that *S*. *tenellus* can distribute in a wide range of salinity (0.7―26.6 PSU), but also that the abundance of this species was greater at low salinity. Furthermore, *S*. *tenellus* has been classified as an “oligohaline neritic” species [42]. Therefore, the estuary sides (D1―M1), where the salinity is particularly diluted by river-discharged water, may allow the *S*. *tenellus* population to maintain its abundance. Interestingly, *S*. *tenellus* was also selected as a “hypereutrophic” species with a high Indval value (78.7%) when the copepod community was grouped by trophic state (Table 3), indicating that the distribution of the species may be associated not only with lower salinity but also with highly eutrophic conditions. In contrast, the species present at high frequencies around the sluice gates could be associated with high-salinity oceanic water. In the classified group of salinity range, *Centropages* spp. were characterized as “polyhaline” species (Table 3). Although the GLM analysis identified the positive effects of salinity on only *C*. *abdominalis*, the species *Centropages* spp., *Tigriopus* spp. or *L*. *rotunda* had no relationship with salinity, despite most of the other species being negatively associated with salinity (Table 4). This means that the abundance of these species was not limited to high salinity. However, because *C*. *abdominalis* was not included among the site-specific species (around the sluice gates), the specific distribution of *Centropages* spp., *Tigriopus* spp. and *L*. *rotunda* could have resulted from their introduction from outside the sluice gates.

The copepod diversity (*H’*) showed seasonal (higher in summer and fall, lower in spring) and spatial gradient differences (higher around the sluice gates, lower around the estuarine sides) (Fig 4), although the effects of water temperature, salinity and Chl *a* on the diversity index were not well explained in the GLM analysis (Table 4). For the seasonal difference, the lower diversity was consistent with lower total abundance, lower richness and lower evenness in spring (Fig 4). This may have resulted particularly from the low evenness associated with the strong dominance exerted by *A*. *hadsonica* (Fig 5). As suggested from the results of the GLM analysis (Table 4), although high water temperature limited total abundance, the greater diversity observed from summer to fall could be associated with low salinity and high Chl *a* (reflecting high phytoplankton biomass): (1) most of the copepod species were negatively affected by salinity, meaning that low salinity allowed the appearance of some species; (2) total abundance would not be largely limited by salinity; (3) higher Chl *a* concentration could increase species richness. For the spatial gradient, the copepod diversity clearly increased with increasing species richness (Fig 4). This trend can be linked to the spatial gradient of salinity (Fig 2). Islam et al. [43] and Tseng et al. [4] found a positive correlation between salinity and copepod diversity in estuarine environments. As mentioned above with regard to the species which occur under specific conditions (*Centropages* spp., *Tigriopus* spp. and *L*. *rotunda*) around the sluice gates, the higher copepod diversity may be associated with the introduction of marine species from the ocean.

To evaluate the applicability of copepods as biological indicators of the condition of the waters in an inlet or bay waters, Ueda [42,44] classified copepods into four ecological terms: “oligohaline neritic,” “eutrophic neritic,” “mesotrophic neritic” and “oligotrophic neritic” from his observations on the abundance and distribution of copepods in Maizuru Bay, Kumihama Bay and Shijiki Bay in Japan. The classification is highly applicable to the copepod community in enclosed waters because it was based not only on salinity level but also on trophic state (associated with phytoplankton biomass) and predation pressure from fishes. According to these ecological terms, the copepods identified in this study can be categorized as follows; oligohaline neritic (*A*. *sinjiensis*, *A*. *pacifica*, *P*. *marinus* and *S*. *tenellus*), eutrophic neritic (*A*. *hudsonica* and *O*. *davisae*), mesotrophic neritic (*Ce*. *abdominals*, *O*. *similis* and *P*. *parvus s*. *l*.) and oligotrophic neritic (*C*. *sinicus*). As shown in the results of the indicator species analysis (Table 3), the species classified into “salinity range” or “trophic state” groups had much in common with the categorization of Ueda. Although the eutrophic-related species *A*. *hudsonica* and *O*. *davisae* were not selected as “eutrophic” or “hypereutrophic” (Table 3), these species were widely distributed in the Saemangeum Reservoir at high abundance, and clearly followed a seasonal change, meaning that the Indval value of each species could be always calculated to be higher if the community were grouped by “salinity range” or “trophic state.” In contrast, as *S*. *tenellus* was selected as “D1―M1,” “oligohaline” and “hypereutrophic” simultaneously, the eutrophic- and low salinity-related species would have the same characteristics. However, in a situation like that of the Saemangeum Reservoir, the species appearing around the estuarine sides or around the sluice gates should have been largely affected by river-discharged freshwater or the oceanic water, respectively. Therefore, the results from the present study can contribute to the characterization of copepod species associated with the effect of river-discharged water (as trophic state) and oceanic water (as salinity), which expands the applicability of copepods as biological indicators in enclosed estuaries.

## Conclusions

The spatiotemporal variation of the composition of the copepod community, in response to different environmental conditions in the Saemangeum Reservoir was analyzed in this study. We determined not only the role which temperature, salinity, and Chl *a* concentration played in structuring copepod community composition but also the species-specific distributional characteristics of the identified copepods in terms of season, spatial gradient, salinity range, and trophic state. The species classified with respect to site, salinity range and trophic state can represent the effect of discharged freshwater and marine-derived seawater, which could be the consequence of eutrophication and gate operation, respectively. The biological indices of copepods described here are expected to be applicable to other enclosed estuaries, where assessment of the impact on ecological conditions of exchanges between freshwater and seawater (linked to alternative consequences between eutrophication and salinization) are required.
